# Anterior portion of the cingulate gyrus: A novel location for transient global amnesia?

**DOI:** 10.1590/S1980-57642014DN81000015

**Published:** 2014

**Authors:** Renata Barbosa Menezes, Edla Renata Cunha Cavalcante, Fernanda Martins Maia, Norberto Anizio Frota

**Affiliations:** 1 Medical Doctor at Northeast Medical Emergencies; 2 Medical Undergraduate of University of Fortaleza, Ceará, Brazil.; 3 Staff Member, Neurology Department, General Hospital of Fortaleza, Ceará, Brazil; 4 Asssitant Professor at the University of Fortaleza, Ceará, Brazil.

**Keywords:** transient global amnesia, cyrus cinguli

## Abstract

Transient global amnesia (TGA) is characterized by abrupt transient loss of anterograde memory, lasting up to 24 hours, and no other focal neurological signs. We report the case of a right-handed 71-year-old female patient who presented temporal-spatial disorientation 5 minutes after ingestion of 1000 ml of iodinated contrast. The patient had mild temporal-spatial disorientation, with significant deficit in anterograde memory. After 12 hours under observation, the patient progressed to gradual improvement and was discharged. A reevaluation after 15 days showed normal cortical functions, score on mini-mental state exam of 30, and unaffected working and recall memory. MRI performed 48 hours after the event showed hypersignal in the diffusion sequence in the anterior portion of the cingulate gyrus, with hypointense signal in MAP/ADC, confirming a finding consistent with TGA. No previous reports in the literature have described the location affected in this patient, rendering it a novel site consistent with this diagnosis.

## Abstract (pt)

Amnésia global transitória (TGA) é caracterizada por perda transitória abrupta de memória anterógrada, com duração de até 24 horas e sem outros sinais neurológicos focais. Relatamos o caso de uma paciente de 71 anos de idade, destra, que apresentou desorientação temporal e espacial depois de 5 minutos após a ingestão de 1.000 ml de contraste iodado. A paciente teve desorientação temporoespacial moderada, com déficit significativo de memória anterógrada. Depois de permanecer 12 horas sob observação, progrediu para melhoria gradual e recebeu alta. A nova avaliação após 15 dias mostrou funções corticais normais, com mini-exame mental de 30, com memória executiva e de evocação preservadas. A ressonância magnética realizada 48 horas após o evento mostrou hipersinal na sequência difusão na porção anterior do giro do cíngulo, com hipossinal em MAPA/ADC, confirmando achado compatível com TGA. Não há relato na literatura descrevendo alteração no mesmo local que esta paciente, tornando-se, portanto, um possível novo local compatível com diagnóstico de TGA.

## INTRODUCTION

Transient global amnesia (TGA) is characterized by abrupt transient loss of anterograde memory, lasting up to 24 hours, absence of focal neurological signs and rare recurrences predominantly in women between 50 and 80 years of age.^1-3^ Some authors report TGA as an event trigged by migraine, Valsalva maneuvers (venous reflux), transient focal ischemia or changes in environmental temperature (hot water bath or cold lake).^2,4^

The method of magnetic resonance imaging (MRI) using diffusion sequence is used to investigate the possible structures affected during the event. A hyperintense signal is typically found, mainly in the hippocampus, but structures such as the amygdala, lenticular nucleus and neocortex were also reported in a study by Nakada et al.^5^ These structures all belong to the limbic system, forming the Papez circuit, which is associated with memory processes.

The case described below presents a typical picture of TGA, but with atypical location on the diffusion MRI in the anterior portion of the cingulate gyrus. To date, there are no reports of the involvement of this structure in investigations of TGA cases by imaging.

## CASE REPORT

MSSB, a 71-year-old, right-handed woman and previously hypertensive was attended at a tertiary hospital in Fortaleza for a routine computed tomography of the abdomen for recurrent abdominal pain. During the preparation phase of the CT, approximately 5 minutes after ingestion of one Liter of iodinated contrast (Telebrix^®^ 20 ml, 1500 ml of water), the patient presented with temporal-spatial disorientation, not remembering the date, or what she was doing there. According to the attendant, the patient had 2 episodes of vomiting in small quantities, and then was taken to the emergency room. At first, her blood pressure was 150 x 90, glucose 96, with no significant findings on physical examination.

On neurological examination, about 1 hour after the onset of symptoms, the patient was alert, remained with mild temporal-spatial disorientation, not identifying the day of the month or week, with significant deficit in anterograde memory for both working and evocation domains. Her memory was tested using the items of the mini-mental state exam (immediate recall, interference followed by evocation) and also by a non-verbal task - 3 objects were shown to the patient and hidden, an interference task was performed and then evocation of the objects placed was tested. The patient's retrograde memory was normal, showing correct recall of data preceding the episode. Praxis, language, and sensitivity were all normal. At the time of the evaluation, the patient's behaviour was completely normal, and insight concerning the deficit was evident. No other focal deficits were detected.

**Figure 1:**
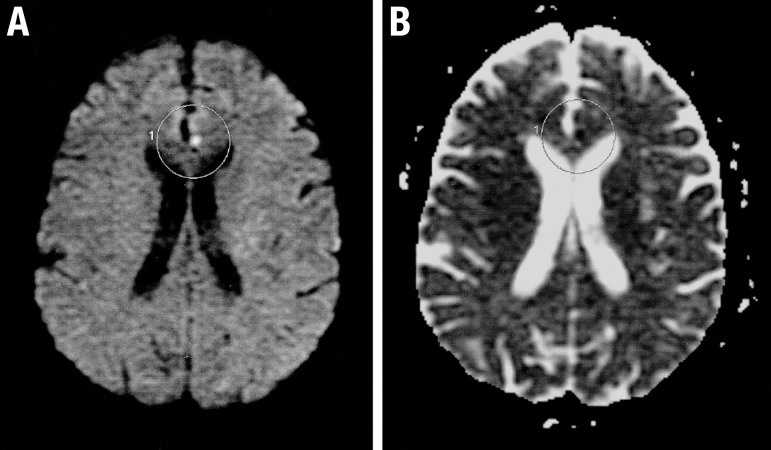
[A] Hyper signal in the diffusion sequence in the anterior portion of cingulate gyrus. [B] Hypointense signal in MAP/ADC in the anterior portion of the cingulate gyrus.

An initial CT scan was obtained and showed no significant abnormalities. MRI performed 48 hours after the event showed a spot of hypersignal on the diffusion sequence in the anterior portion of the cingulate gyrus, with hypointense signal in MAPA / ADC, which is consistent with restriction of water diffusion (Figure 1). An extended EEG revealed no epileptiform activity. A complete lab workup was performed, on which all blood counts, electrolytes, cardiac enzymes, hepatic enzymes, urine analysis tested normal

The hypothesis of irreversible dementia was excluded due to acute onset. Some of the differential diagnoses considered were infection of the central nervous system, prolonged complex partial seizures, transient epileptic amnesia (TEA), head injury / cerebral contusion, psychogenic amnesia, subarachnoid haemorrhage, stroke in the hippocampus and thalamus, intoxication and drug intake.

After remaining under observation in the emergency room, she showed gradual improvement of the symptoms and was discharged about 12 hours after the start of the symptoms. A reevaluation of the patient 15 days after the event showed intact cortical functions, with mini-mental state exam score of 30, and unaffected working memory and recall. A new MRI was performed and showed no abnormalities in the anterior portion of the corpus callosum.

## DISCUSSION

Some hypotheses have been raised about the pathophysiology of TGA, among them the lack of an internal jugular vein. Lewis^6^ proposed in 1998 that situations increasing blood reflux, such as the Valsalva maneuver, those of vasoconstrictors and peripheral immersion in ice water, could generate a venous congestion in the region of the hippocampus followed by subsequent ischemia. In 2001, Akkawi et al.^7^ showed the presence of a retrograde venous flow component in 46.7% (n=30) of patients using duplex ultrasound during the Valsalva maneuver. Our patient most probably had some degree of transient increase in intracranial pressure due to a Valsalva maneuver secondary to the vomiting episodes and therefore abnormalities in the venous flux can also be considered.

There is worldwide discussion about the connection of migraine with aura and TGA. Authors explain that there are decreased levels of glutamate, promoting a depression of brain electrical activity that initially manifests with tissue hyperperfusion followed by hypoperfusion, causing the migraine. This mechanism could possibly be the same as TGA and might explain some results showing that patients with a history of migraine are more likely to have an episode of TGA.^1-4^ In our case, the patient had no complaints of headache, thereby ruling out this mechanism.

Another cause of TGA is the use of contrast for imaging. There is one case report that documented a probable TGA during a Valsalva maneuver after administration of saline to perform a transesophageal echocardiogram in a patient with patent foramen ovale.^8^ Iodinated contrast agents used in coronary angiography was also reported as precipitating TGA. In the case in question, three hours after contrast administration, the patient developed disorientation in time and space, but without focal neurological signs. An explanation for this phenomenon is that toxicity of iodine causes platelet dysfunction which leads to a decrease in cerebral blood flow.^9^

The occurrence of neurotoxicity after administration of iodinated contrast can be found in the literature, mostly associated with intra-arterial administration.^10^ In these cases, cerebral edema and small ischemic areas were described, akin to that found in this patient. However, there was no edema evidenced and the literature does not report any association of neurotoxicity with oral iodinated contrast, making this hypothesis less likely to explain our patient's clinical presentation. Furthermore, patients with underlying brain conditions, impaired kidney function, those who have received a large contrast dose or prolonged exposure to contrast media are at the greatest risk for developing contrast neurotoxicity, where the patient reported met none of these conditions.^11,12^

The diagnostic criteria for TGA are presence of anterograde amnesia, which is witnessed by an observer; no clouding of consciousness or loss of personal identity; cognitive impairment limited to amnesia; no focal neurological or epileptic signs; no recent history of head trauma or seizures; resolution of symptoms within 24 hours; mild vegetative symptoms (headache, nausea, dizziness) might be present during the acute phase.^4^

It was important to analyze the hypothesis of a stroke as a differential diagnosis for this case. The fronto-polar artery, a branch from the anterior cerebral artery, is responsible for the irrigation of the area altered in the first MRI of this patient. Although a strong hypothesis in the beginning, an embolic/thrombotic mechanism was excluded since the control images did not disclose the previous alteration, showing complete resolution in a short space of time and without any deficits.

Regarding the location, the anterior cingulate gyrus is linked to a variety of behavior manifestations. Functional neuroimaging studies in humans show that it plays an important role in emotional memory content.^13,14^ Recent studies with mice demonstrate that the anterior cingulum participates in the memory consolidation process.^15^

TGA studies report the change, for almost 94% of cases, classically in the hippocampal CA1 portion,^16^ which is best seen 48-72 hours after the start of the event, when the sensitivity of the technique in the sequence of diffusion becomes more evident.^17-20^ Other locations have also been described including the thalamus, caudate nucleus, corpus callosum, retrosplenial cortex, lenticular nucleus, and frontal lobe.^21^ However, the anterior cingulum is not cited in any published reports thus becoming a novel location to be considered a site consistent with a diagnosis of TGA.
